# NOA1 Functions in a Temperature-Dependent Manner to Regulate Chlorophyll Biosynthesis and Rubisco Formation in Rice

**DOI:** 10.1371/journal.pone.0020015

**Published:** 2011-05-23

**Authors:** Qiaosong Yang, Han He, Heying Li, Hua Tian, Jianjun Zhang, Liguang Zhai, Jiandong Chen, Hong Wu, Ganjun Yi, Zheng-Hui He, Xinxiang Peng

**Affiliations:** 1 Key Laboratory of Plant Functional Genomics and Biotechnology, Education Department of Guangdong Province, South China Agricultural University, Guangzhou, China; 2 Institute of Fruit Tree Research, Guangdong Academy of Agricultural Sciences, Guangzhou, China; 3 College of Agriculture, South China Agricultural University, Guangzhou, China; 4 Department of Biology, San Francisco State University, San Francisco, California, United States of America; University of Georgia, United States of America

## Abstract

*NITRIC OXIDE-ASSOCIATED1* (*NOA1*) encodes a circularly permuted GTPase (cGTPase) known to be essential for ribosome assembly in plants. While the reduced chlorophyll and Rubisco phenotypes were formerly noticed in both *NOA1-*supressed rice and *Arabidopsis*, a detailed insight is still necessary. In this study, by using RNAi transgenic rice, we further demonstrate that NOA1 functions in a temperature-dependent manner to regulate chlorophyll and Rubisco levels. When plants were grown at 30°C, the chlorophyll and Rubisco levels in *OsNOA1*-silenced plants were only slightly lower than those in WT. However, at 22°C, the silenced plants accumulated far less chlorophyll and Rubisco than WT. It was further revealed that the regulation of chlorophyll and Rubisco occurs at the anabolic level. Etiolated WT seedlings restored chlorophyll and Rubisco accumulations readily once returned to light, at either 30°C or 15°C. Etiolated *OsNOA1*-silenced plants accumulated chlorophyll and Rubisco to normal levels only at 30°C, and lost this ability at low temperature. On the other hand, de-etiolated *OsNOA1*-silenced seedlings maintained similar levels of chlorophyll and Rubisco as WT, even after being shifted to 15°C for various times. Further expression analyses identified several candidate genes, including *OsPorA* (NADPH: protochlorophyllide oxidoreductase A), *OsrbcL* (Rubisco large subunit), *OsRALyase* (Ribosomal RNA apurinic site specific lyase) and *OsPuf4* (RNA-binding protein of the Puf family), which may be involved in OsNOA1-regulated chlorophyll biosynthesis and Rubisco formation. Overall, our results suggest OsNOA1 functions in a temperature-dependent manner to regulate chlorophyll biosynthesis, Rubisco formation and plastid development in rice.

## Introduction

Nitric oxide (NO) functions not only in plant defense responses, but also in the regulation of various plant developmental processes, including germination, root growth, vascular differentiation, stomatal closure, and flowering [1]. The mechanism underlying nitric oxide (NO) synthesis has been well established in animals, and depends primarily on a heme-flavoprotein NO synthase (NOS). NOS catalyzes the conversion of arginine to citrulline and NO in the presence of NADPH and oxygen [2]. NOS1 in *Arabidopsis* (AtNOS1) was first characterized as a potential nitric oxide synthase, which shares homology to a hypothetical snail NOS or NOS partner [3]. NO levels in *Atnos1*-knockout mutants were significantly reduced compared to WT plants [3], [4], [5], and moreover, the yellowish phenotype of the *Atnos1* mutants was partially rescued by the application of NO donor compounds [3], [6], [7]. The *NOS1*-deficient plants also showed decreased NO accumulation in response to ABA, salicylic acid, salinity, and elicitor treatments [4], [5], [8]–[10].

Studies so far have yielded conflicting data regarding the true function of NOS1. In some studies it was observed that the accumulation of NO in response to different hormones or oxidative stresses was similar in *NOS1-*deficient and WT plants [11]–[13]; and contrastingly different NO responses in the mutants to different treatments have been detected, even under similar experimental conditions [5]. It is thus possible that NOS1 is involved in NO production in response to some stimuli but not to the others [1]. In addition, a role for NOS1 unrelated to NO synthesis was suggested by the observation that not all of the phenotypes noticed in the mutants could be rescued by NO supplementation [7]. Direct evidence came about recently by using recombinant NOS1 systems, where NOS activity was not observed [14]–[16], leading to the later renaming of AtNOS1 to NO-associated protein 1 (AtNOA1) [14]. It is currently more accepted that NOA1 affects NO production in an indirect manner [1].

AtNOA1 was identified and annotated as an open reading frame of 1686 bp, corresponding to a 561 amino acid protein that shares no sequence homology to animal NOSs. It belongs to a circularly permuted GTPase (cGTPase) family [16], [17]. In bacteria and some eukaryotes, this family of GTP-binding proteins is associated with the RNA/ribosome binding function [16]–[18]. Loss of function of AtNOA1 causes a chlorotic phenotype and an up-regulation of methylerythritol phosphate (MEP) pathway enzyme levels [7]. In the rice genome, only one homologous gene was identified: *Os02g0104700* (*OsNOA1*). OsNOA1 was recently shown to be a functional homolog of AtNOA1/RIF1 required for ribosome biogenesis [1], [7] and plastid function [19]. However, how exactly NOA1 plays a role in plastid function is still poorly understood.

While the chlorophyll and Rubisco decreasing phenotypes were formerly noticed in either *NOA1-*supressed rice or *Arabidopsis*, a detailed insight is still lacking. In this study, by using RNAi transgenic rice plants, we further demonstrate that NOA1 functions in a temperature-dependent manner to regulate chlorophyll and Rubisco levels. It was further revealed that the regulation of chlorophyll and Rubisco occurs at the anabolic level, and that reduced chlorophyll and Rubisco levels may result in defective plastids.

## Materials and Methods

### Plant materials

*Oryza sativa* (japonica cultivar-group) Zhonghua 11 was used for the physiological study and for constructing the transgenic plants.

### Growth condition and treatments

Pre-germinated seeds were grown for 10d in Kimura B complete nutrient solution, which was renewed every 3 days [20] under a greenhouse condition [average temperature of 30/25°C (day/night), relative humidity 60–80%, photosynthetically active radiation 600–1000 µmol·m^−2^ s^−1^ and photoperiod of 14 h day/10 h night] or in a phytotron chamber [for the experiments of gradient temperatures, photosynthetically active radiation 80 µmol·m^−2^ s^−1^, relative humidity 65%, photoperiod 12 h light/12 h dark, 22/22°C, 26/26°C or 30/30°C (light/dark); for the experiments of gradient light intensities, temperature 22/22°C(light/dark), relative humidity 65%, photoperiod 12 h light/12 h dark, 80, 150 or 350 µmol·m^−2^ s^−1^]. The second leaf from the top was detached at about 2 cm, and then stored at −75°C for subsequent analyses.

### Construction of *OsNOA1*-silenced plants

To generate the *OsNOA1* interference construct, a 446 bp sequence ranging from 404 to 849 bp of the ORF cDNA was amplified by RT-PCR with an upstream primer 5′- TTGTGAGCTCTGCTATGTGGGAGATGC-3′ and a downstream primer 5′- ACAGGATCCCCAACATTTGCTGAACC-3′. A skeleton RNAi vector termed pYLRNAi.5 was kindly provided by Dr. Yao-Guang Liu (College of Life Sciences, South China Agricultural University, China). The vector harbors two multi-cloning sites (MCS) so as to be able to more conveniently insert the target sequence in sense vs. antisense orientations. The cloned *OsNOA1* cDNA fragment was first inserted in a sense orientation at MCS1 between *Sac*I and *Bam*HI. Both PCR with the specific primers and restriction enzyme cutting verified that the fragment had been correctly inserted into the vector. This first round ligated vector was then used as a template to amplify a second sequence with two unique restriction sites at the ends (RNAi-*Mlu*I: 5′-CACCCTGACGCGTGGTGTTACTTCTGAAGAGG-3′, RNAi-*Pst*I: 5′-ACTAGAACTGCAGCCTCAGATCTACCATGGTCG-3′). The second sequence was subsequently cloned at MCS2 between *Pst*I and *Mlu*I, thereby resulting in an opposite orientation as the sequence in MCS1. Restriction enzyme cutting showed that the second target fragment had been correctly inserted into the vector. Finally, sequencing analyses further confirmed the correct orientation and the cDNA identity [99% identical to that reported in the NCBI (*Os02g0104700*)]. The constructed interference vector named as *pYLRNAi-Ubi-OsNOA1* was then transformed into rice callus by *Agrobacterium*-mediated infection (strain EHA105). T0 lines were first analyzed by Southern blot. T1 seeds from T0 lines with a single T-DNA insertion were grown to produce T2 seeds. Further screening with hygromycin-resistance identified the homozygous plants.

### Measurements of photosynthesis, stomatal conductance and transpiration

Leaf net photosynthetic rates, stomatal conductance and transpiration rates were measured by a portable gas analysis system, LI-COR 6400 with a light-emitting diode light source (LI-COR Inc., Lincoln, NE). The second leaf from the top was used for the analyses. The measurement conditions were set as follows: leaf temperature 25°C, photon flux density 800 µmol·m^−2^ s^−1^, humidity 65%, and CO_2_ concentration 0.038%. All of the above measurements were conducted between 11:00 and 14:00 on the day of measurement.

### Subcellular localization in protoplasts

The cDNA of *OsNOA1* (1.7 kb) was amplified by RT-PCR from *Oryza sativa* (japonica cultivar-group) and confirmed by sequencing. The following primers were used in the PCR to create *Sal*I and *Sac*I sites: 5′-TATGTCGACCTCCTCCTGCTCCTAGT-3′ and 5′- GTCGAGCTCCAGTAATGCCATTTAGGT-3′. *p35S-OsNOA1-GFP* constructs were generated by cloning the 1.7 kb cDNA into the *Sal*I-*Sac*I site of pUC19-GFP vector. After polyethylene glycol (PEG)-mediated transformation of *Arabidopsis* leaf-derived and rice stem-derived protoplasts [21], the fluorescent signals of GFP and chloroplast were detected using an Olympus fluorescent microscope, and all images acquired were analyzed using Image-Pro software (Olympus, Tokyo, Japan).

### Electron microscopy

Fresh leaf tissue was cut into blocks (1 mm^3^) with a sharp blade and prefixed in Karnovsky's fixative (3% paraformaldehyde, 4% gluteraldehyde, 0.1 M phosphate buffer, pH 7.2) for 4 h at 4°C, washed 3 times in 0.1 M phosphate buffer (pH 7.2), postfixed for 1 h in 1% OsO_4_, transferred back into phosphate buffer and then washed 3 times. After dehydration through an acetone series, the samples were rinsed in 50% Epon812 epoxy resin overnight, and subsequently transferred into 100% epoxy resin overnight before polymerization at 40°C for 24 h and 60°C for 24 h. Ultrathin sections (70-80 nm) were cut using a diamond knife on a LEICA EM UC6 microtome and then stained with uranyl acetate for 20 min, and counterstained with lead citrate for 10 min. The sections were examined and photographed on a Philip Fei-Tecnai 12 transmission electron microscope.

### Microarray analysis

Whole leaves and stems from either WT or line 20 grown at 22°C for 6d were pooled together in liquid N_2_ for microarray analysis. RNA was isolated using Trizol Reagent (Invitrogen). Affymetrix GeneChip Rice Genome Arrays were used, and the genechip analyses were performed at CapitalBio Corporation (Beijing, China). The transcripts whose expressions were changed by at least 2-fold were defined to be differentially expressed. Results for gene transcripts changed by at least 2-fold can be found in Table S1 available at *PLoS ONE* online. All microarray data is MIAME compliant and the raw data from this article have been deposited in the ArrayExpress database (http://www.ebi.ac.uk/arrayexpress/) under accession number E-MEXP-3136.

### Quantitative RT-PCR

Total RNA was isolated using TRIZOL reagents (Invitrogen). One microgram of RNA was used as a template for first-strand cDNA synthesis using ReverTra Ace (Toyobo, Osaka, Japan) with random hexamers according to the manufacturer's instructions. Primer pairs for real-time quantitative PCR (see Table S3 online) were designed using Primer Premier 5.0 (Premier Biosoft, Palo Alto, USA). The PCR reaction consisted of 10 µL of 2×SYBR Green PCR Master Mix (Toyobo), 200 nM primers, and 2 µL of 1:40-diluted template cDNA in a total volume of 20 µL. No template controls were set for each primer pair. Real-time PCR was performed employing the DNA Engine Option 2 Real-Time PCR Detection system and Opticon Monitor software (Bio-Rad, USA).

### Protein extraction, detection of Rubisco abundance and western blot analyses

Protein was extracted by homogenizing 0.1 g of fresh leaves in 0.5 ml of 20 mM phosphate buffer (pH 7.5). The homogenate was centrifuged at 12,000×g for 15 min. Equally loaded proteins (10 µg) were fractionated on 12.5% SDS-PAGE gels, which were stained with CBB R-250. The gels were scanned by WinRHIZO LA 1600+ scanner (Epson, Japan) at a resolution of 300 dpi. All images were analyzed using Quantity One software (Bio-Rad, USA). For the western blot analysis of OsNOA1, proteins from equal fresh weight were loaded in each lane, separated by 10% SDS-PAGE, transferred onto a nitrocellulose membrane, and finally immunodetected using rabbit anti-OsNOA1 serum.

### Measurement of chlorophyll content

Chlorophyll was extracted from leaves with mixed solution (45% acetone, 45% ethanol and 10% distilled water), and the content was then determined spectrophotometrically at 645 and 663 nm according to Lichtenthaler [22].

## Results

### Generation of the *OsNOA1*-silenced lines

**Figure 1:**
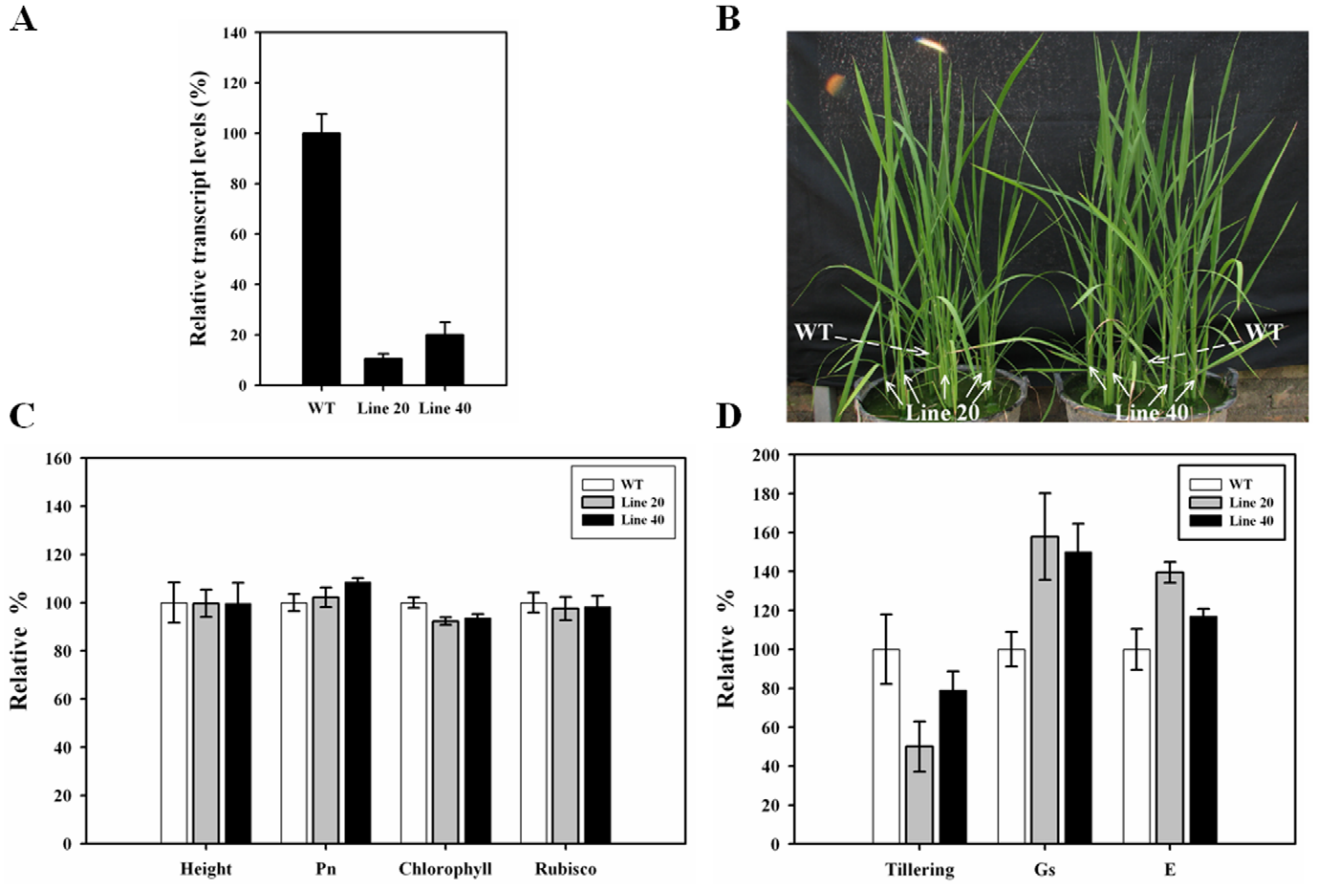
The phenotypes of *OsNOA1*-silenced lines 20 and 40 under normal natural growth conditions. (A) Transcriptional expression of *OsNOA1* (n = 6); (B) Images of rice growth; (C) Relative height (n = 24), photosynthesis rates (Pn) (n = 16), chlorophyll (n = 3) and Rubisco (n = 3) in the silenced and WT plants; (D) Relative tillering (n = 24), stomatal conductance (Gs) (n = 16) and transpiration rates (E) (n = 16). The second leaf from the top was sampled for the analyses. The data are the means ±SD of n replicates and representative of at least three independent experiments.

Examination of the rice genome identified a single gene (*Os02g0104700*) that is homologous to *AtNOA1/RIF1*, which we named *OsNOA1*. *OsNOA1* has an open reading frame (ORF) of 1644 bp that encodes a protein of 547 amino acids with 62% similarity to AtNOA1/RIF1. The protein has a conserved YqeH domain (137–340aa) and a predicted GTPase domain. A sequence ranging from 404 to 849 bp of the ORF was amplified by RT-PCR to be used to construct the RNAi vectors. The constructs were then transformed into WT rice via *Agrobacterium*. Southern blot analyses identified several independent T_0_ lines with a single T-DNA insertion, including lines 20 and 40 used in this study. The T_1_ seeds from these two lines were grown to produce T_2_ seeds, from which homozygous lines were identified through a hygromycin-resistance screen. Real-time PCR analyses confirmed that *OsNOA1* transcript levels are indeed significantly reduced by about 90% and 80% for lines 20 and 40, respectively (Fig. 1A). Therefore, we used these two independent homozygous lines for further functional analyses.

### Phenotypes

Under normal natural conditions, the *OsNOA1*-silenced plants had similar growth rates to WT (Fig. 1B, C). The contents of chlorophyll and Rubisco were slightly lower in the silenced plants than those in WT (Fig. 1C). No significant differences were detected in the photosynthetic rate (Pn) and the ultrastructure of plastids (Fig. S3). In contrast, the tiller number was reduced by 50% and 21% for lines 20 and 40, respectively, while the stomatal conductance and transpiration rate were increased (Fig. 1D). The increased stomatal conductance and transpiration may be due to reduced NO in the *NOA1*-suprressed plants [3], [16]. *OsNOA1* fused with a GFP reporter gene was transfected into both *Arabidopsis* leaf protoplasts and rice stem protoplasts. The results demonstrated that OsNOA1 is localized to the plastids of rice (Fig. S1), in agreement with previous reports that the OsNOA1-EYFP fusion protein is targeted to chloroplasts in transgenic *Arabidopsis* plants [19].

**Figure 2:**
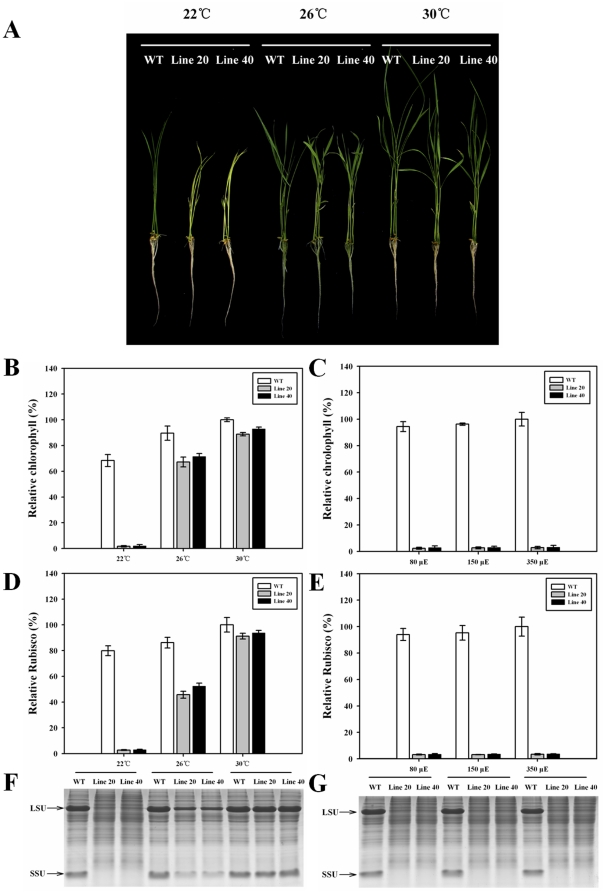
Growth, chlorophyll and Rubisco as affected by temperature and light intensities in Os*NOA1*-silenced lines. Germinated seeds were grown in growth chambers at different temperatures or various light intensities for 10d. (A) The morphological phenotypes for lines 20, 40 and WT; (B, D, F) Relative content of chlorophyll and Rubisco in lines 20 and 40 at different temperatures under 80 µmol·m^−2^ s^−1^ light intensity; (C, E, G) Relative content of chlorophyll and Rubisco in lines 20 and 40 under different light intensities at 22°C. The second leaf from the top was sampled for the analyses. The data are means ±SD of 3 replicates and representative of at least three independent experiments.

It has been reported that *Atnoa1* knockout mutants display dwarf and yellow leaf phenotypes under normal conditions [3], [7], [16]. Interestingly, as addressed above, we did not see the same phenotypes in our *OsNOA1*-silenced rice plants under the normal natural growth conditions. However, dwarf and yellow leaf phenotypes were observed in our *OsNOA1*-silenced plants when the growth temperature was decreased (Fig. 2A).

### Temperature-dependent changes in chlorophyll, Rubisco and plastids

We next examined in detail the effects of various temperatures and light intensities on chlorophyll and Rubisco accumulations in the *OsNOA1*-silenced plants. At 30°C, only minor reductions were detected in the contents of chlorophyll and Rubisco in the silenced plants as compared to WT (Fig. 2B, D, F). Significant differences occurred when the growth temperature was decreased to 26°C; and only trace amounts of chlorophyll and Rubisco could be detected in the silenced plants at 22°C (Fig. 2B, D, F; Fig. 5C, D). In order to confirm the temperature-dependence of the NOA1 function, we also tested the *Atnoa1*-knockout *Arabidopsis* in this respect. As shown in Fig. S2, at 22°C the levels of chlorophyll and Rubisco were only slightly lower in the mutants than those in WT, and a lower temperature (12°C) markedly enhanced the chlorotic phenotype in the mutants, with Rubisco decreased to a very low level in the mutants. In contrast, different light intensities had no obvious impact on the abundance of chlorophyll and Rubisco in the silenced plants at 22°C (Fig. 2C, E, G), indicating that *OsNOA1* regulates accumulation of chlorophyll and Rubisco independent of light intensity.

**Figure 3:**
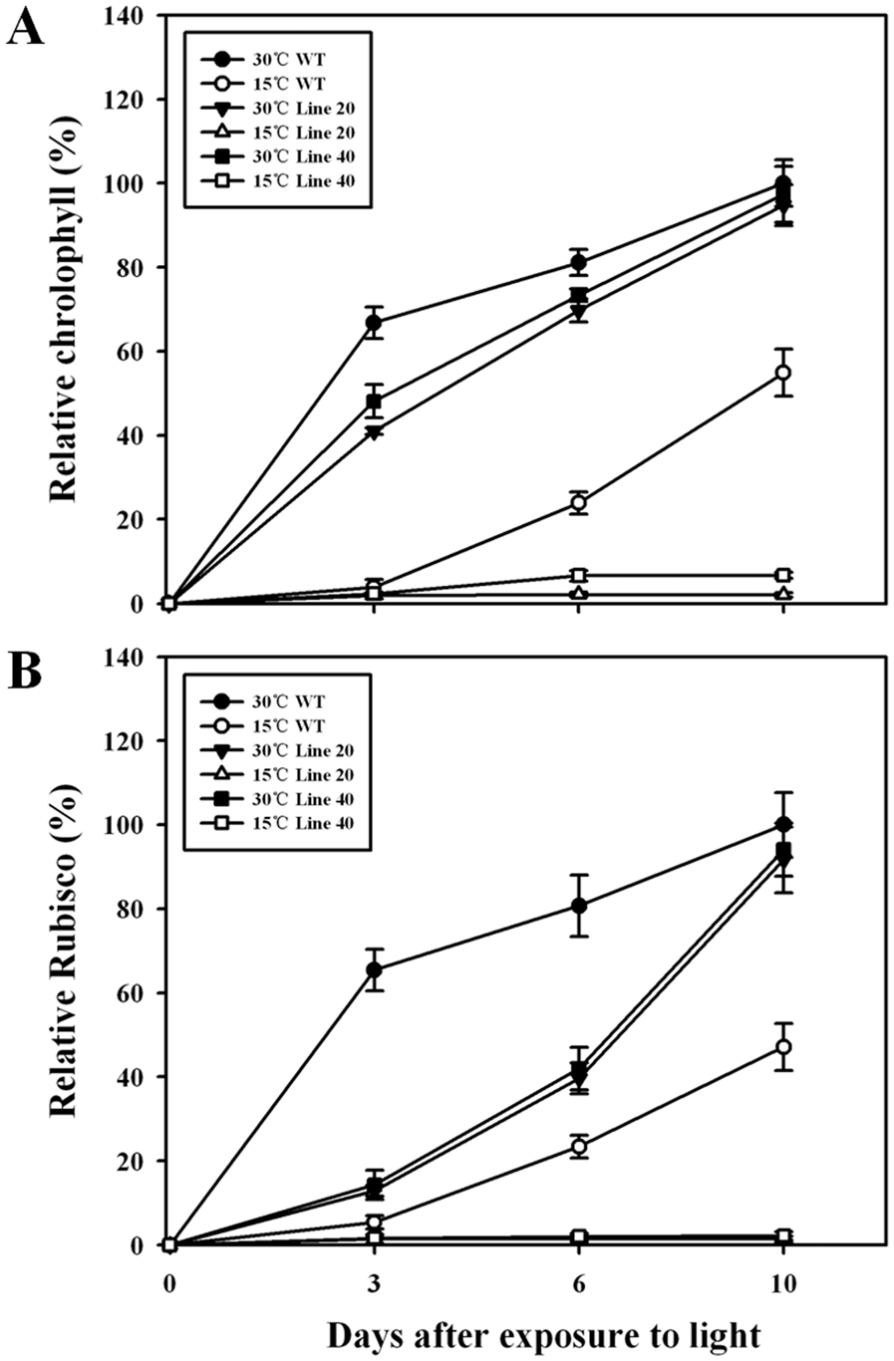
Accumulation of chlorophyll (A) and Rubisco (B) in etiolated silenced seedlings after exposure to light. Germinated seeds (lines 20, 40 and WT) were grown in a growth chamber at 28°C in the dark for 4d, then the etiolated seedlings were exposed to light at either at 30°C or 15°C for various time (0, 3, 6 and 10 d). The second leaf from the top was sampled for the analyses. The data are means ±SD of 3 replicates and representative of at least three independent experiments.

**Figure 4:**
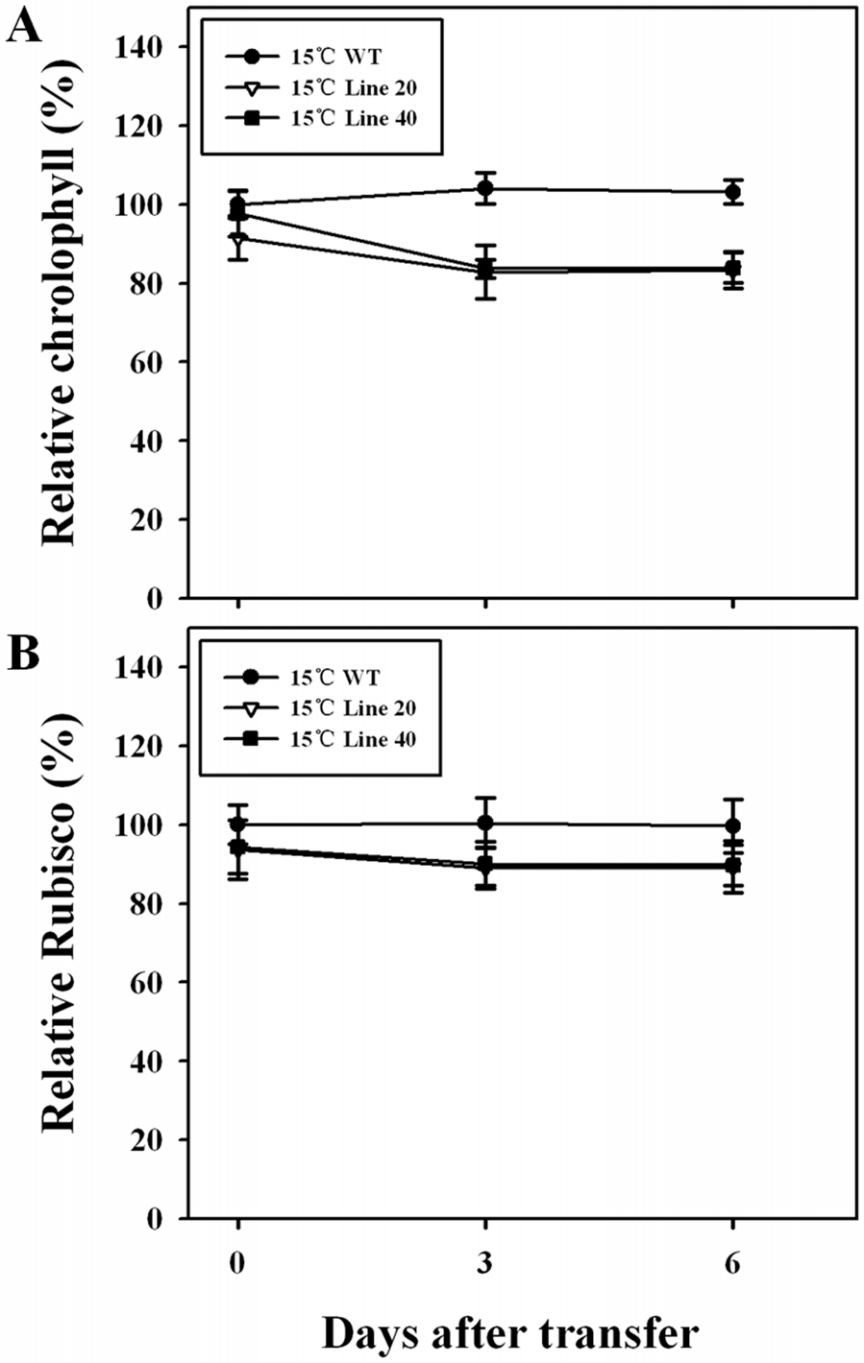
Changes in the accumulated chlorophyll and Rubisco under low temperature. Germinated seeds (lines 20, 40 and WT) were grown in a growth chamber at 30°C and under 80 µmol·m^−2^ s^−1^ light intensity for 10d, then the seedlings were transferred to 15°C under the same light intensity for various times (0, 3, 6d). Leaves that had already fully expanded before transfer were sampled for the analyses. The data are means ±SD of 3 replicates and representative of at least three independent experiments.

When the etiolated seedlings were transferred to light at 30°C, both *OsNOA1*-silenced and WT plants turned green readily, with chlorophyll and Rubisco continuously accumulated. Similar amounts of chlorophyll and Rubisco were detected at 10 d after transfer in the silenced plants relative to WT (Fig. 3). If the etiolated seedlings were transferred to 15°C, the silenced plants failed to accumulate chlorophyll and Rubisco, in contrast to WT that was able to accumulate appreciable amounts of chlorophyll and Rubisco at the lower temperature (Fig. 3). If the silenced seedlings were first grown at 30°C for 10d and then transferred to 15°C, those leaves that had already fully expanded could maintain similar amounts of chlorophyll and Rubisco after transfer (Fig. 4), while those newly-emerged leaves after transfer were yellowish, just like the phenotype of the silenced seedlings that were continuously grown at the lower temperature (Fig. 2A). In addition, it was found that Rubisco accumulation lagged behind chlorophyll. For instance, the chlorophyll level in the silenced seedlings recovered to about 45%, 72% and 95% relative to WT, at 3d, 6d and 10d, respectively, while Rubisco was restored to 14%, 41% and 93% under the same condition (Fig. 3).

**Figure 5:**
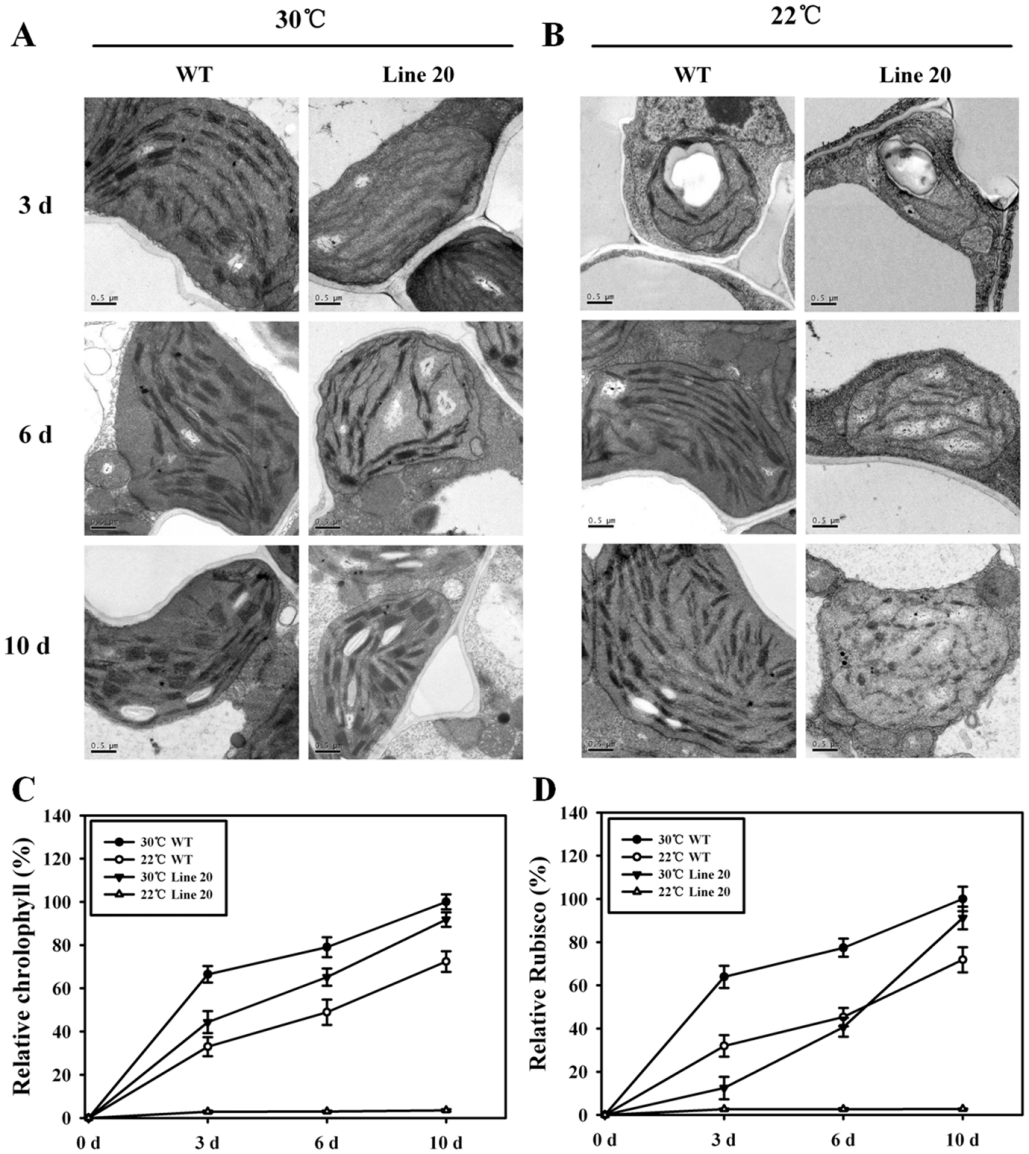
Changes in plastid ultrastructure in relation to the accumulation of chlorophyll and Rubisco in the silenced plants. Germinated seeds (line 20 and WT) were grown in growth chambers at either 30°C (A) or 22°C (B) and under 80 µmol·m^−2^ s^−1^ light intensity for various times (3, 6, 10d). The newest leaf (3, 6d) or the second leaf from the top (10d) was sampled for the observation of ultrastructure of plastids (A and B) and determination of chlorophyll and Rubisco content (C and D). The ultrastructure was observed by a Philip Fei-Tecnai 12 transmission electron microscope. Bars = 0.5 µm. The data are means ±SD of 3 replicates and representative of at least three independent experiments.

As shown in Fig. 5A, obvious differences in plastid ultrastructure could be seen between the *OsNOA1*-silenced and WT plants grown at 30°C for 3d. WT had thickly stacked thylakoid membranes, while the silenced plants had vague thylakoid membranes. As the plants grew up at this temperature, the difference gradually became smaller and almost disappeared at 10d. Clearly, a positive correlation exists between chlorophyll and Rubsico accumulations and plastid development (Fig. 5A vs. Fig. 5C). In contrast, little differences in plastid ultrastructure could be seen between the *OsNOA1*-silenced and WT plants grown at 22°C for 3d (Fig. 5B). As the plants grew up at this lower temperature, the plastids in WT gradually developed and finally matured, while the plastids in the silenced plants were degraded gradually and almost totally dissolved at 10d (Fig. 5B). It was meanwhile noticed that, at 22°C, chlorophyll and Rubisco remained at a very low level throughout the time course (Fig. 5C, D).

The above data collectively indicate that the decreased chlorophyll and Rubisco levels are ultimately due to their impaired biosynthesis or formation, rather than to an increase in degradation. This decrease in production may then affect plastid development.

### Transcriptional responses to *OsNOA1* suppression

**Figure 6:**
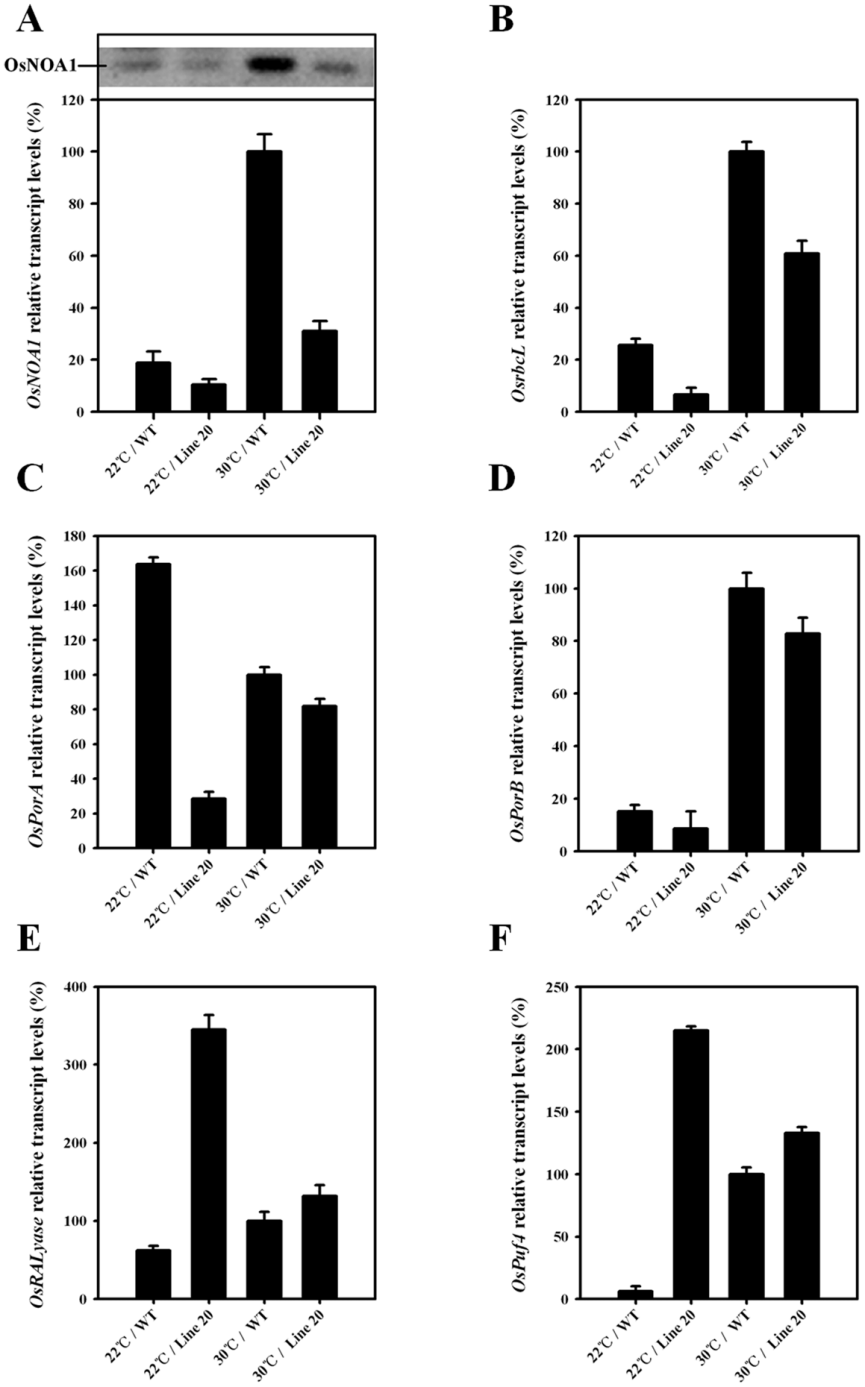
Expressions of some genes in response to *OsNOA1* silencing at either 22°C or 30°C. Germinated seeds (line 20 and WT) were grown at either 22°C or 30°C for 10d. The whole leaves and stems were sampled for various analyses (real-time PCR for all the genes listed; additional western-blot for OsNOA1 as in A). The data are means ±SD of 3 replicates and representative of two independent experiments. GenBank/EMBL accession numbers: *OsNOA1*, NM_001052149; *OsrbcL*, NP_039391; *OsPorA*, NM_001060809; *OsPorB*, NM_001071490; *OsPuf4*, NM_001073324 and *OsRALyase*, NM_001053416.

To further understand the possible reasons behind the decreased chlorophyll biosynthesis and Rubisco formation, a cDNA microarray analysis was performed to screen for candidate genes that are affected by the *OsNOA1* silencing. The assay identified a total of 2292 transcripts that showed more than a 2-fold change in expression (Table S1 and S2). Among these genes, 1362 were up-regulated and 930 were down-regulated (Table S1). Two genes, *NADPH: protochlorophyllide oxidoreductase A* (*PorA*, *Os04g0678700*) and *chlorophyllide a oxygenase* that are responsible for chlorophyll biosynthesis, were highly down-regulated in *OsNOA1*-silenced plants at 22°C, whereas the genes involved in chlorophyll metabolism were not significantly affected (Tables S1 and S2). *RbcL, PsbC*, *PsbD,ferredoxin-NADP^+^ reductase* and *ATPase subunit ε* were down-regulated and the other genes involved in the photosystems, carbon fixation and the TCA cycle were not altered (Table S1 and S2). A translational repressor gene *OsPuf4* (*Os12g048890*), and *OsRALyase* (*Os02g0493100*) encoding a putative ribosomal RNA apurinic site specific lyase, were greatly up-regulated (Table S1) in the silenced plants at 22°C. Subsequent real-time PCR analyses confirmed that the expressions of *OsPorA*, *OsrbcL, OsPuf4* and *OsRALyase* were indeed dramatically altered at 22°C, and restored to normal when the growth temperature was increased to 30°C (Fig. 6B–F). In addition, expression of our target gene *OsNOA1* was highly suppressed at both mRNA and protein levels in WT at 22°C, such that the expression difference between the silenced and WT plants were even larger at 30°C than at 22°C (Fig. 6A), seemingly inconsistent with the phenotypes (Fig. 2A).

## Discussion

### Temperature-dependent phenotypes and possible reasons behind

Most recently, Liu *et al*. [19] observed that *OsNOA1-*silenced rice plants had yellowish leaves and decreased Rubisco abundance when grown in a temperature-controlled green house (30°C day/25°C night). It was also noticed that when *Arabidopsis* was grown at constant 22 or 24°C (16h light/8h dark) the newly-emerged young leaves of *Atnoa1*-knockout mutants displayed a yellow phenotype though mature leaves had only a slight reduction in chlorophyll [6], [7]. In our current study, however, under a normal natural condition (average 30°C day/average 25°C night) or at constant 30°C in a growth chamber (12h light/12h dark), *OsNOA1-*silenced plants had only slightly lower levels of chlorophyll and Rubisco than WT plants, without visual chlorotic phenotypes (Figs. 1B and 2A). Clearly, quantitative differences occurred between these experiments. We consider that these inconsistencies could be caused by the ambient temperature differences. In our study, 30°C during the day is an average estimated value, but the maximum could reach as high as 35°C at noon. It has been noticed that an even higher temperature (*e. g.* constant 35°C) was able to completely abolish the minor differences that were observed at constant 30°C (data not shown). This temperature-dependence was subsequently examined in detail. We observed that, as growth temperature was decreased, the differences became increasingly larger, with chlorophyll and Rubisco dramatically reduced in the silenced plants at low temperature (Figs. 2 and 3). Similar phenotypes were also observed in the *Atnoa1*-knockout *Arabidopsis* (Fig. S2). To the best of our knowledge, such a temperature-dependent regulation of NOA1 function has not been addressed so far.

It is an intriguing question as to how temperature affects NOA1 function, with special regard to the regulation of chlorophyll and Rubisco. Temperature-sensitive plant mutants and variants were previously reported, but a common type is those that show normal phenotypes at low temperature and mutant phenotypes at high temperature [23]–[25]. The type like *NOA1-*silenced rice observed in this study, i. e. permissive at high temperature and restrictive at low temperature, has been also noticed more recently. For instance, a mutant designated as *tds* (a recessive temperature dependent shooty mutant of *Nicotiana tabacum* L) displays thick and narrow leaves with abnormal mesophyll cells at 21°C. A higher temperature at 30°C reverses this mutant phenotype and leads to formation of normal leaves and restoration of apical dominance [26]. A *phan* mutant has arrested growth at 15°C, but shifting the mutant from 15°C to 25°C allows meristem function to resume [27]. An *Arabidopsis* mutant, called *chilling sensitive5* (*chs5*), contains a missense mutation in a conserved amino acid of 1-deoxy-d-xylulose 5-phosphate synthase (DXP synthase). The mutant displayed a temperature-sensitive phenotype, being normal at 22°C but chlorotic at 15°C [28]. In a pepper mutant named *Sy-2*, the true leaf at 20°C is irregularly shaped with a smaller area, but is almost normal when the growth temperature is above 24°C [29], [30].

Mechanisms underlying the temperature-dependent phenotypes, particularly where low temperature is restrictive, are poorly understood thus far. One possible explanation for such an effect may be that NOA1 itself directly serves as an effecter to maintain or even up-regulate the tolerance of plants to low temperature, and loss of its function would increase sensitivities to low temperature, thereby resulting in reduced chlorophyll and Rubisco in plants. If it is true, NOA1 would be of evolutionary significance and particularly important for the tropically-originated species, such as rice, to adapt to cold environments. It was further thought that NO may have mediated the regulation by NOA1 since NO was shown to be involved in cold tolerance [31], [32] and reduced NO production was observed in *NOA1*-deficient plants [3], [31]. However, it was detected that the supplementation of NO failed to restore the reduced chlorophyll and Rubisco (Fig. S4) [19], ruling out the above possibility. In addition, reactive oxygen species (ROS) were usually increased in *NOA1*-deficient plants [6], [8]. Persistent increase in ROS may attenuate the tolerance to low temperature leading to a faster degradation of chlorophyll and Rubisco [33], [34]. We have shown, however, that the reduced chlorophyll and Rubisco are ultimately due to the impaired chlorophyll biosynthesis and Rubisco formation, rather than to their degradation (Fig. 3 and 4). Thus, a direct involvement of ROS in the reduced chlorophyll and Rubisco seems also less likely, and whether indeed NOA1 functions in the regulation of low-temperature tolerance needs to be further investigated. A second possibility has been previously raised by other authors [26], [28] that a kind of protein exists to be able to compensate for the absence of the gene product at high temperatures but not at low temperature. This compensatory protein could be either homoeologous (same family member) or distinct. As described above, since only one gene copy is identified for *OsNOA1* in the rice genome, the proposed protein is more likely to be distinct from OsNOA1. It is highly interesting to identify the proposed compensatory protein and those linking components between NOA1 and chlorophyll/Rubisco turnover. In this study, by using microarray and real-time PCR analyses, we preliminarily identified several possible linking genes, i. e. *OsPorA*, *OsrbcL*, *OsRALyase* and *OsPuf4* which are known to be involved in chlorophyll and Rubisco anabolism. But, how NOA1 regulates these genes and what those interaction proteins are remains to be understood.

*OsNOA1* expression in WT plants was found to be highly down-regulated at low temperature (Fig. 6A), and similar results were also observed in *Arabidopsis* [31]. This result is puzzling because the difference in *OsNOA1* expression between the silenced and WT plants at 22°C was even smaller than that at 30°C (Fig. 6A), which is seemingly inconsistent with the phenotypes (Fig. 2). It is not clear as to why no significant phenotypes occur in WT at 22°C, even though *OsNOA1* expression is also suppressed (Fig. 6A). We here propose a possibility that *OsNOA1* expression is redundant and that there is a threshold for its function. At low temperature, this threshold for WT plants might be lower than that for silenced plants. This notion is based on the evidence that certain proteins, presumably including the compensatory one, were more suppressed in the silenced plants than in WT plants, particularly at low temperature (see more discussions below) [19].

### Chlorophyll and Rubisco are regulated by OsNOA1 at the anabolic level

It was previously proposed that the yellow leaf phenotype in *NOA1*-suppressed plants was caused by the oxidative stress-induced degradation of chlorophyll resulting from impaired ribosome biogenesis due to loss of function of NOA1 [16], [19]. It is also possible that the oxidative stress could be directly elicited by the suboptimal growth temperature. But generally, the critical minimum temperature for rice shoot elongation ranges from 7 to 16°C and that for root elongation from 12 to 16°C [35]. Here we show several lines of evidence to indicate that OsNOA1 regulates chlorophyll biosynthesis and Rubisco formation rather than their degradation: (i) The etiolated WT plants restored chlorophyll and Rubisco accumulations readily once returned to light at either 30°C or 15°C, while the etiolated silenced seedlings could normally accumulate chlorophyll and Rubisco only at 30°C and lost the ability at the low temperature (Fig. 3); (ii) Once the silenced plants had accumulated sufficient chlorophyll and Rubisco (in the light at 30°C), their levels were not significantly reduced even after they were transferred to 15°C for various times (Fig. 4); (iii) Expression analyses further point to such a possibility. *PorA* and *OsrbcL*, a gene for chlorophyll biosynthesis [36] and for the Rubisco large subunit, respectively, were heavily suppressed at 22°C but almost normal at 30°C in the *NOA1*-silenced plants, and all the genes detected for chlorophyll catabolism were not significantly affected (Table S2). The other two genes, *OsRALyase* and *OsPuf4*, were highly up-regulated at 22°C but returned to normal levels at 30°C (Fig. 6E, F and Table S1). OsRALyase was identified as an rRNA apurinic site-specific lyase (RALyase) [37], [38], which cleaves phosphodiester bonds at the depurinated site produced by ribosome-inactivating protein and diminishes the residual elongation activities of depurinated ribosomes. In support, it was detected that certain rRNA species needed for ribosome assembly were indeed reduced in the silenced rice plants at 22°C (data not shown), which was also noticed by Liu *et al*. [19]. Pufs are able to suppress protein translation by binding their specific target to mRNAs [39]. These results further indicate that the formation of either Rubisco or some other proteins may be retarded at the translational level in the silenced plants at 22°C. There have also been studies showing that NOA1 is required for the correct formation of 70S ribosomes and stability of 30S subunits in *Arabidopsis* [16], [40] and in rice [19]. Overall, it appears highly likely that OsNOA1 regulates chlorophyll biosynthesis and Rubisco formation rather than their degradation in rice plants.

### Plastid development and NO production in relation to chlorophyll and Rubisco

Chlorophyll, Rubisco and plastids were all affected in the silenced plants at low temperature (Figs. 2, 3 and 5). Thus, we are curious about whether the three of them are mechanistically interrelated. It was noticed that chlorophyll was accumulated faster than Rubisco (Figs. 3 and 5), so there is a possibility that Rubisco formation is subsequently regulated by chlorophyll accumulation. But, this notion seems to be negated by the data from both Paddock *et al.* [41] and Bevins *et al*. [42], who showed that, in some mutants of *Arabidopsis* where chlorophyll was mostly lost, Rubisco large subunit levels were still indistinguishable from that in WT plants. Thus, chlorophyll and Rubisco could be independently regulated by OsNOA1. Loss of any inclusion, including chlorophyll, Rubisco and NO, could be simply accounted for by pleiotropic effects of defective plastids [7], [31]. However, our time-course dynamic observation in this study indicated that the deficiency of chlorophyll and Rubisco might have acted as the cause, rather than the effect, of the defective plastids (Fig. 5). This notion is supported by some previous experiments. For instance, a *POR* mutant that was retarded in chlorophyll biosynthesis developed a seedling-lethal *xantha* phenotype at the cotyledon stage and possessed defective plastids [41]; a sweetclover chlorophyll-deficient mutant also showed abnormal development of plastids [42]. Indeed, our *OsNOA1-*silenced seedlings grown at 22°C displayed similar plastid ultrastructure as the above mutants [41], [42], where those authors held the view that the defective plastid ultrastructure in the mutants might be caused by the reduced chlorophyll biosynthesis [41]–[43]. In addition, a total of 28 plastid ribosomal proteins were substantially reduced in *OsNOA1*-silenced rice [19], such a reduction in plastome-encoded proteins was also considered to be the cause of defective plastids [7]. Collectively, the possibility exists that altered chlorophyll and Rubisco might have affected plastid development.

NOA1 was once considered as a NO synthase in plants, but it is currently more accepted that NOA1 affects NO production in an indirect manner [1]. According to Moreau *et al*. [16], decreased NO production in *Atnoa1* knockout mutants may result from increased production of reactive oxygen species (ROS), since interaction of the elevated ROS with NO reduces the amount of NO. Increased ROS could simply be caused by the pleiotropic effect of defective plastids [6], [7], [8], [16] and/or disrupted chlorophyll biosynthesis [44]–[46]. It has been reported that the ‘nonphotoactive’ Pchlide (protochlorophyllide *a*), due to its dissociation from the POR active site, may serve as a photosensitizer for the formation of singlet oxygen [44]–[46]. A heavy down-regulation of *PorA* in our silenced rice, therefore, may likely elicit ROS production in such a similar manner. Indeed, several studies have observed increases in ROS production in *NOA-*suppressed plants [3], [8].

## Supporting Information

Figure S1 Subcellular localization of OsNOA1-GFP in rice and *Arabidopsis* protoplasts. Rice stem and Arabidopsis leaf protoplasts transfected with p35S-OsNOA1-GFP constructs were observed under fluorescent microscopy. The green color image reflects the green fluorescence of OsNOA1-GFP and the red indicates the autofluorescence of chlorophyll in chloroplasts. Images were then merged to show overlapping green and red fluorescence in yellow. Bars = 5μm.
(TIF)

Figure S2 The phenotypes (A) and SDS-PAGE (B) of *Atnoa1* mutants and WT grown at either 22°C or 12°C under 80 μmol·m^-2^ s^-1^ light intensity for 16d.
(TIF)

Figure S3 The plastid ultrastructure of line 20 and WT grown under normal natural growth conditions. The plants were grown under normal natural growth conditions until the booting stage, then the second leaf from the top was sampled for plastid observation. The ultrastructure was observed by a Philip Fei-Tecnai 12 transmission electron microscope. Bars noted in the bottom of each image.
(TIF)

Figure S4 Effects of the NO donor SNP on the decreased chlorophyll and Rubisco in *OsNOA1 -*silenced rice. Germinated seeds (lines 20, 40 and WT) were grown on Kimura B complete nutrient agar supplemented with different concentrations of SNP (10, 50, 100μM) in a growth chamber at 22°C and under 80 μmol·m^-2^ s^-1^. The SNP solution was renewed every 3 days. The whole leaves and stems of 6-old-day seedlings were sampled for the analyses. The data are means ±SD of 3 replicates and representative of two independent experiments.
(TIF)

Table S1 Transcripts at least 2-fold changed in *OsNOA1*-silenced line 20 compared with WT.
(XLS)

Table S2 Microarray analysis of gene expressions in response to *OsNOA1* silencing.
(XLS)

Table S3 Primer pairs for real-time quantitative PCR.
(XLS)
