# Supplementary figures and images for: NOA1 Functions in a Temperature-Dependent Manner to Regulate Chlorophyll Biosynthesis and Rubisco Formation in Rice
Source: PLoS One. 2011 May 23;6(5):e20015. doi: 10.1371/journal.pone.0020015 (PMC3100308; doi:10.1371/journal.pone.0020015)

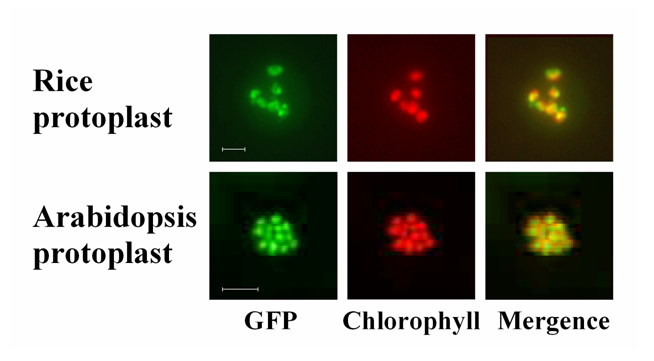

Supplement: Figure S1 — Subcellular localization of OsNOA1-GFP in rice and Arabidopsis protoplasts. Rice stem and Arabidopsis leaf protoplasts transfected with p35S-OsNOA1-GFP constructs were observed under fluorescent microscopy. The green color image reflects the green fluorescence of OsNOA1-GFP and the red indicates the autofluorescence of chlorophyll in chloroplasts. Images were then merged to show overlapping green and red fluorescence in yellow. Bars = 5μm. (TIF) [file pone.0020015.s001.tif]

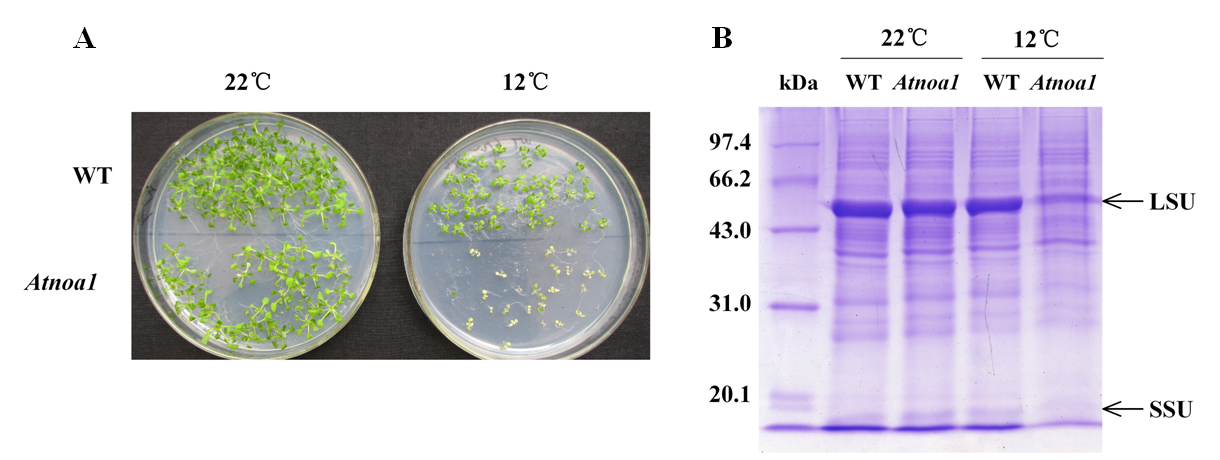

Supplement: Figure S2 — The phenotypes (A) and SDS-PAGE (B) of Atnoa1 mutants and WT grown at either 22°C or 12°C under 80 μmol·m-2 s-1 light intensity for 16d. (TIF) [file pone.0020015.s002.tif]

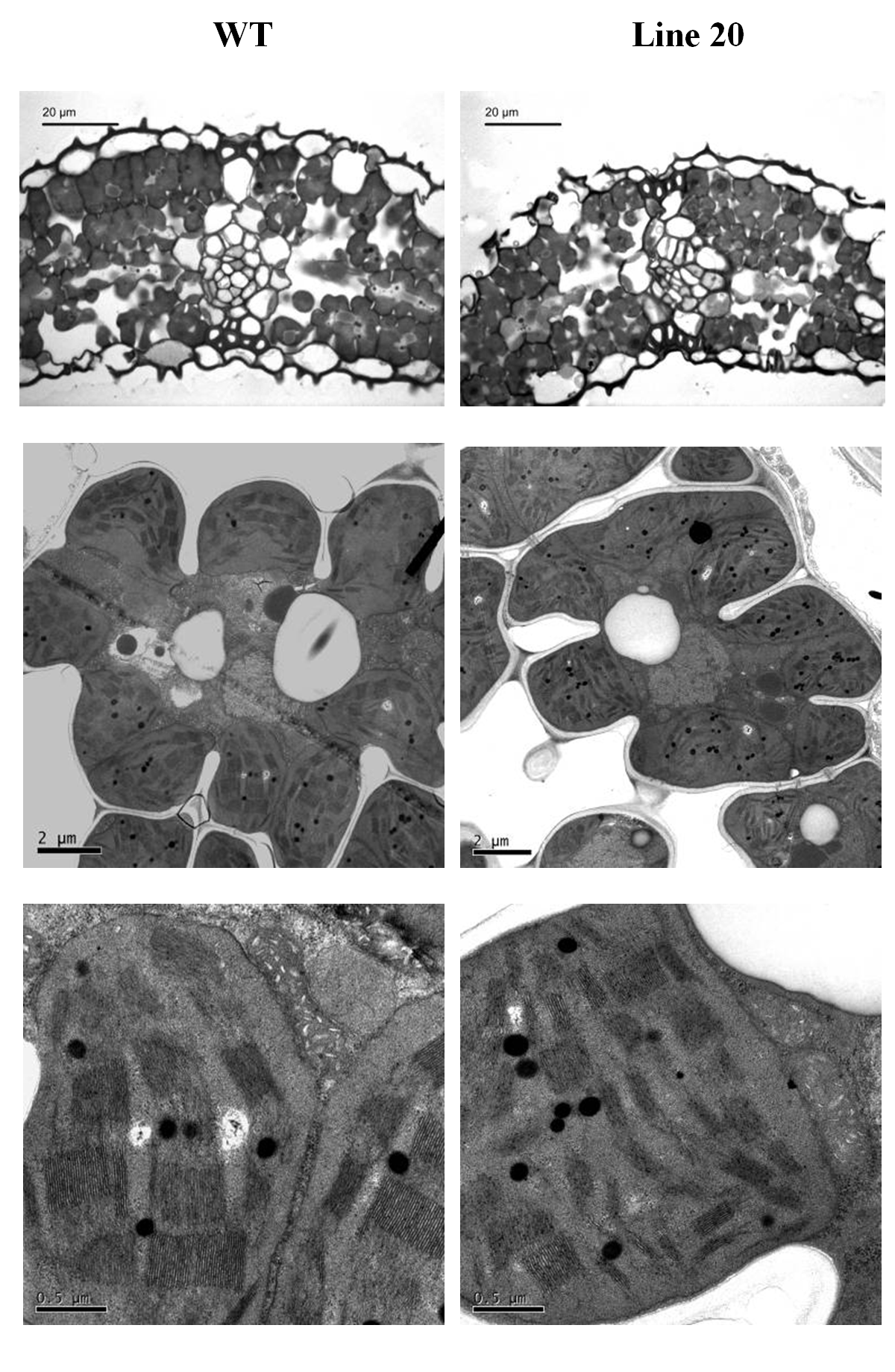

Supplement: Figure S3 — The plastid ultrastructure of line 20 and WT grown under normal natural growth conditions. The plants were grown under normal natural growth conditions until the booting stage, then the second leaf from the top was sampled for plastid observation. The ultrastructure was observed by a Philip Fei-Tecnai 12 transmission electron microscope. Bars noted in the bottom of each image. (TIF) [file pone.0020015.s003.tif]

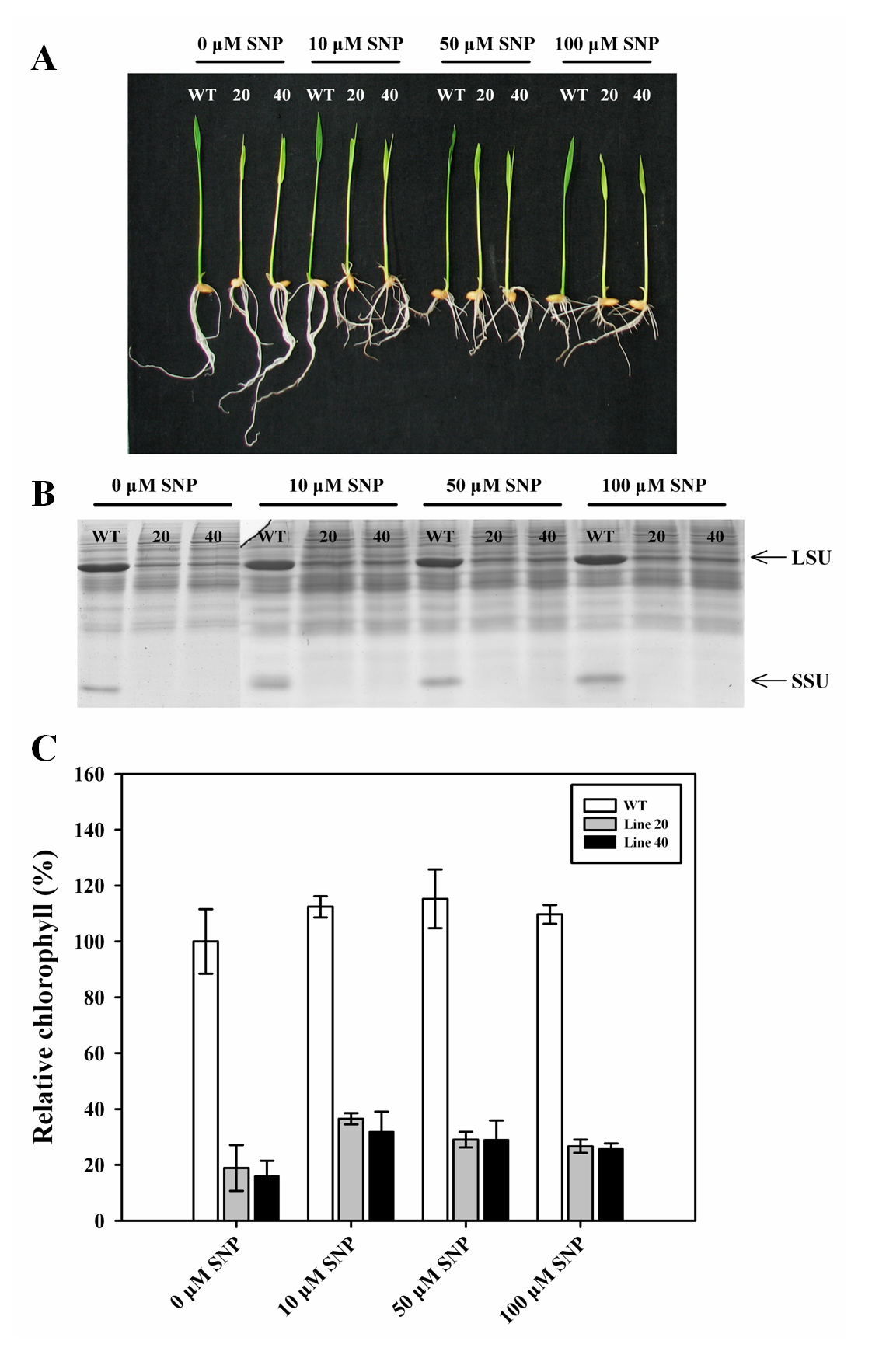

Supplement: Figure S4 — Effects of the NO donor SNP on the decreased chlorophyll and Rubisco in OsNOA1 -silenced rice. Germinated seeds (lines 20, 40 and WT) were grown on Kimura B complete nutrient agar supplemented with different concentrations of SNP (10, 50, 100μM) in a growth chamber at 22°C and under 80 μmol·m-2 s-1. The SNP solution was renewed every 3 days. The whole leaves and stems of 6-old-day seedlings were sampled for the analyses. The data are means ±SD of 3 replicates and representative of two independent experiments. (TIF) [file pone.0020015.s004.tif]
